# Oligomerization of 10,16-Dihydroxyhexadecanoic Acid and Methyl 10,16-Dihydroxyhexadecanoate Catalyzed by Lipases

**DOI:** 10.3390/molecules18089317

**Published:** 2013-08-05

**Authors:** M. Beatriz Gómez-Patiño, Julia Cassani, María Eugenia Jaramillo-Flores, L. Gerardo Zepeda-Vallejo, Georgina Sandoval, Manuel Jimenez-Estrada, Daniel Arrieta-Baez

**Affiliations:** 1 Instituto Politécnico Nacional - ENCB, Carpio y Plan de Ayala S/N, Col. Casco de Santo Tomas, México, D.F., CP 11340, Mexico; 2 Departamento de Sistemas Biológicos, Universidad Autónoma Metropolitana Unidad Xochimilco, Calz. del Hueso No.1100, Col. Villa Quietud, México, D.F., CP 04960, Mexico; 3 CIATEJ, Av. Normalistas 800, Guadalajara, Jalisco 42270, Mexico; 4 Departamento de Productos Naturales, Instituto de Química, UNAM. México, D.F. CP 04510, Mexico; 5 Instituto Politécnico Nacional - CNMN, Calle Luis Enrique Erro s/n, Unidad Profesional Adolfo López Mateos, Col. Zacatenco, México D.F., CP 07738, Mexico

**Keywords:** lipases, oligomerization, natural monomer, tomato cuticle

## Abstract

The main monomer of tomato cuticle, 10,16-dihydroxyhexadecanoic acid (10,16-DHPA) and its methyl ester derivative (methyl-10,16-dihydroxyhexadecanote; methyl-10,16-DHHD), were used to study their oligomerization reactions catalyzed by five lipases: *Candida antarctica* lipase B (CAL-B), *Rhizomucor miehei* lipase (RM), *Thermomyces lanuginosus* lipase (TL), *Pseudomonas cepacia* lipase (PCL) and porcine pancreatic lipase (PPL). For 10,16-DHPA, optimum yields were obtained at 60 °C using toluene and 2-methyl-2-butanol (2M2B) as solvent, while for methyl-10,16-DHHD the bests yields were obtained in toluene and acetonitrile. Both reactions leaded to linear polyesters according to the NMR and FT-IR analysis, and there was no data indicating the presence of branched polymers. Using optimized conditions, poly(10,16-DHPA) and poly(methyl-10,16-DHHD) with Mw = 814 and Mn = 1,206 Da, and Mw = 982 and Mn = 860 Da, respectively, were formed according to their MALDI-TOF MS and ESI-MS data. The self-assembly of the polyesters obtained were analyzed by AFM.

## 1. Introduction

The synthesis of aliphatic polyesters continues to be of significant interest in biomedical applications (drug delivery systems, wound closure and healing products, and surgical implant devices) [1,2,3] and some applications that include food containers, soil retention sheeting, agriculture film, waste bags and the use as a packaging material in general [4]. However, most of these polyesters are semi-crystalline, hydrophobic, and lack free functional group(s) for further structural modification [5,6,7]. One way to address these drawbacks is the introduction of such functional groups. The presence of free functional groups such as hydroxyl and carboxylate in polyesters can efficiently increase their hydrophilicity and degradability, as well as modulate their mechanical, thermal, chemical, and biologic properties [5,6,8]. Furthermore, these functional groups could allow conjugation of a variety of bioactive molecules to obtain novel biomaterials with diverse applications. Poly(malic acid) and polyesters containing L-malic acid units are attractive because of their remarkable advantages in temporary therapeutic applications [9]. Many hydroxyl- or carboxyl-functional pendant groups along the macromolecular chains of these polymers could facilitate covalent prodrug attachment. Kline [10] and Uyama [11] have prepared hydroxyl-functionalized materials from trifunctional alcohols and divinyl acid derivatives. Kumar [12] has demonstrated the direct condensation of a diacid with either sorbitol or glycerol to make similar hydroxyl-functionalized materials. While these are examples of useful materials, they are not completely linear. Sorbitol yielded 85% 1,6 regioselectivity, while glycerol only yielded 66% 1,3 regioselectivity [12]. Most examples of these functionalized materials have been prepared via ring-opening polymerization (ROP) of cyclic esters, cyclic anhydrides, derivatized lactones or lactides and include materials with amino, carboxyl, and hydroxyl groups [13,14]. Despite their utility, there remains a need to design new polymeric materials with more variation in solubility, crystallinity, polarity, and reactivity. This need for an expanded set of properties has driven research in the area of preparing aliphatic polyesters based on the use of biodegradable and renewable sources.

Since biopolymers are biodegradable and the main productions are obtained from renewable resources such as agroresources, they represent an interesting alternative route to common non-degradable polymers. In this regard, biomass represents an abundant carbon-neutral renewable resource for the production of biomaterials. Many of these renewable resource polymers can also be rendered biodegradable under the appropriate conditions [15].

Cutin is the most abundant polyester in Nature and constitutes the framework of the protective cuticle of stems and leaves of higher plants [16,17,18,19]. Despite of different efforts to get the monomers presents in cutin, the complexity and differences between different species has made difficult the analysis of specific monomers in polymerization reactions [20,21,22]. Recently, *cis*-9,10-epoxy-18-hydroxyoctadenoic acid was isolated in high yield from outer birch bark (*Betula verrucosa*), and polymerized by CAL-B to get epoxy-functionalized polyesters with high molecular weight [23]. Tomato cutin is one of the most studied cuticles, and different monomers have been isolated and characterized [20,24,25]. However, only a few investigations on polymerization reactions using tomato monomers have been reported [26,27].

Lipase-catalyzed polymerization may sometimes allow straightforward synthesis strategies for polyesters, which are difficult to prepare by more conventional polymerization processes. In the field of cell-free enzyme catalyzed polyester synthesis, the enzyme most often studied due to its high catalytic activity for an extraordinarily diverse range of monomeric substrates is Lipase B from *Candida antarctica* (CALB) [28,29]. This enzyme, immobilized on Lewatit PMMA beads (N435), is commercially available from Novozymes (Baggsverd, Denmark). CAL-B has been extensively used to produce commercial aliphatic polyesters mainly through condensation polymerization of aliphatic dicarboxilic acids with diols, transesterification reaction of diesters with diols, polymerization of hydroxy acids, and ring-opening polymerization of lactones [30,31].

Our interest in the synthesis of aliphatic polyesters is focused on the possibility to produce linear, hydroxyl functionalizable polyesters through a polymerization method that must be versatile enough to allow for the facile preparation of a variety of different materials for a wide range of biomedical applications. Thus, we are reporting for the first time the polymerization of 10,16-dihydroxy-hexadecanoic acid (10,16-DHPA), the main monomer of tomato cutin, and its methylated ester (methyl-10,16-dihydroxyhexadecanoate, methyl-10,16-DHHD) mediated by different lipases. The resulting products were fully characterized by NMR, FTIR, MALDI-TOF MS, ESI MS and AFM analysis.

## 2. Results and Discussion

10,16-Dihydroxyhexadecanoic acid (**1**), the main monomer present in the tomato cuticle, was isolated using reported protocols [20,25]. The compound was identified using NMR analysis and comparing the results with those previously reported [32,33]. The presence of the hydroxyl in position 10 was identified using MS, according with our previous mass analysis of this important monomer [33].

### 2.1. Enzymatic Polyesterification Studies of the 10,16-DHPA

**Scheme 1:**
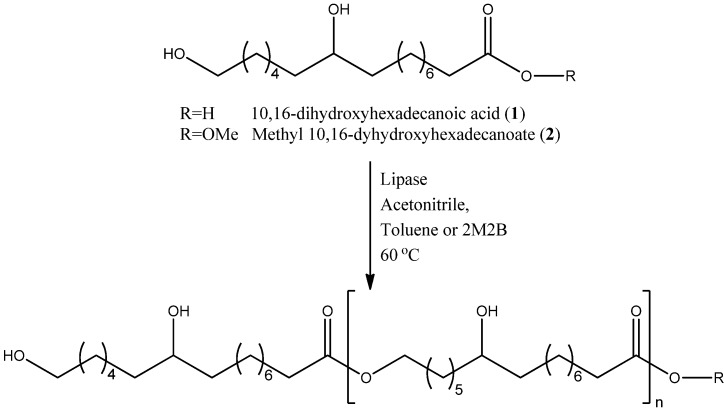
Reaction of 10,16-dihydroxyhexadecanoic acid (10,16-DHPA, **1**) or methyl-10,16-dihydroxyhexadecanoate (methyl-10,16-DHHD, **2**) with lipases.

*Candida antartica* lipase B (Novozyme 435) has been used to efficiently polymerize long-chain aliphatic ω-hydroxy acids [23,29,34]. In this regard, CAL-B was initially used to analyze the polyesterification of the 10,16-DHPA (Scheme 1). The monomer was not soluble in hexane and acetonitrile at 60 °C and it was only soluble in acetonitrile at 70 °C. However, no reactions were detected in this solvent. CAL-B-mediated reactions of 10,16-DHPA were only detected in toluene and 2M2B, even when the monomer was soluble in DMF as well. After 24 h there were no changes in the reaction according to the HP-TLC (chloroform:methanol, 9:1, v:v) analysis. Once the initial conditions for the polymerization reactions were established, CAL-B, PCL, PPL, RM and TL were assayed using toluene and 2M2B as a solvent.

Related long-chain linear aliphatic polyesterifications are difficult to accomplish by more conventional polymerization process, and more of these reactions have been done with lipases, more specifically with CAL-B. In order to analyze their efficiency with these monomers, five commercial lipases were assayed and according with Table 1, only CAL-B, RM and TL gave good yields and the optimal temperature for the lipase-catalyzed reactions was 60 °C (Table 2).

Weight average molecular weight (Mw) and number average molecular weight (Mn) of the polyesters obtained showed that CAL-B was more effective for the polyester reaction of the 10,16-DHPA (Table 3) even when the NMR and FT-IR analysis did not show differences in the polyesters obtained. It has been reported that molecular sieves can help increase the degree of polymerization (DP) and the polydispersity index (Mw/Mn)in polymerization reactions [35]. However, in this study the reaction time was only a slightly faster when molecular sieves were used. Recently, different studies have shown that the use of molecular sieves does not improve the yield and/or the degree of polymerization [36], and the results in this work are in agreement with them. It is important to note that the excess of water in the lipase solution is responsible for the depolymerization reaction, and low molecular weights could be achieved. So, when molecular sieves were added to the solution to remove the excess water, the polymerization reaction was slightly improved but it seems that the water responsible for the depolymerization reaction remains in the enzyme creating an equilibrium without improving the molecular weights.

**Table 1:** Comparison of the polyester reaction of 10,16-DHPA mediated by lipases in toluene and 2M2B at 60 °C.

Lipase	Toluene	2M2B
CAL-B	80 ± 0.5	75 ± 0.9
TL	65 ± 1.0	65 ± 1.2
RM	64 ± 1.9	40 ± 0.7
PPL	35 ± 1.5	25 ± 0.5
PCL	35 ± 0.9	40 ± 1.8

Results correspond to the products yields.

**Table 2:** Comparison of the polyester reaction of 10,16-DHPA mediated by lipases at 60 °C or 75 °C.

Lipase	Toluene	
	60 °C	75 °C
CAL-B	80 ± 0.9	75 ± 0.5
TL	65 ± 0.8	55 ± 1.5
RM	65 ± 0.7	70 ± 1.0
PPL	35 ± 0.5	40 ± 1.7
PCL	34 ± 1.9	40 ± 1.1

Results correspond to the products yields.

**Table 3:** Molecular weight of the lipase-catalyzed polyesterifications of **1** and **2**.

Polyester	Monomer	Enzyme	Solvent	Mn	Mw	pd	DP
3	1	Cal-B	Toluene	814	1206	1.13	2.96
3	1	RM	Toluene	771	863	1.12	2.86
3	1	TL	Toluene	769	857	1.13	2.97
4	2	CAL-B	Toluene	860	982	1.14	3.19
4	2	CAL-B	Acetonitrile	653	707	1.08	2.42
3	1	CAL-B	2M2B	565	570	1.00	2.10
4	2	CAL-B	2M2B	605	648	1.06	2.25

Reactions were done at 60 °C for 24 h. Number average molecular weight (Mn), Weight average molecular weight (Mw), polydispersity index (pd or Mw/Mn) and degree of polymerization (DP) were calculated with PolyTools 1.0 (Bruker Daltonics).

Successful polyesterifications of aliphatic hydroxyacids using immobilized CAL-B have been reported [29]. Among them, 16-hydroxyhexadecanoic acid showed a relatively high reactivity in a series of reactions of these compounds. However, there were large differences in the molecular weight between them and 10,16-DHPA. Polymerizations οf ω-hydroxyacids with chain lengths of C-16, C-12, and C-10 gave products with DPavg ~ 105 and Mw/Mn of 1.5 by 24 h [29]. However, for the polyesterification products of 10,16-DHPA, the DP was 2.9 and Mw/Mn of 1.1 by 24 h. This behavior could be attributed to the presence of a hydroxyl in position 10 which gives to the molecule an angle of 114.3° (SCHRÖDINGER^®^ Jaguar for ChemBio 3D Ultra, CambridgeSoft, Portland, OR, USA) modifying the linearity of the palmitic acid (PA).

**Figure 1:**
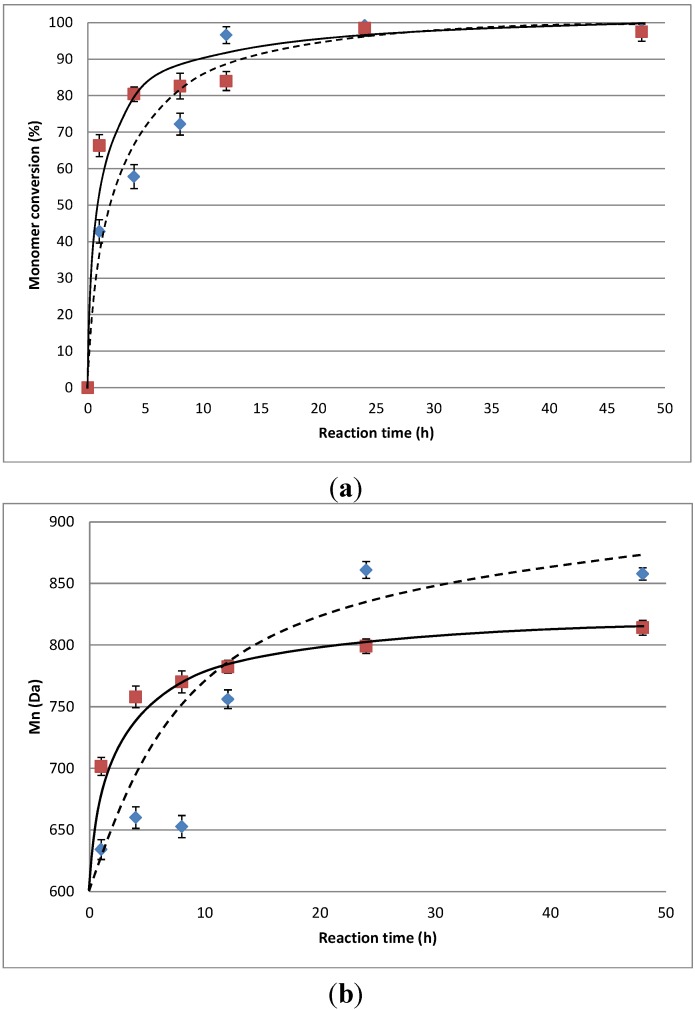
(**a**) CALB-catalyzed polycondensations of 10,16-dihydroxyhexadecanoic acid (10,16-DHPA) (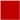) and methyl-10,16-dihydroxyhexadecanoate (methyl-10,16-DHHD) (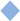) in toluene at 60 °C and (**b**) Extent of chain growth as a function of time of the reaction of 10,16-DHPA (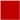) and methyl-10,16-DHHD (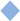). The error bars were calculated from triplicate runs.

Conversion of 10,16-DHPA was followed through an HPLC-ELSD analysis. According to Figure 1a, the enzyme uses the monomer efficiently at 8 h (82%) and it is completely gone at the 24 h (93%). This is in agreement with the analysis of the products (Figure 1b). ESI^−^ MS (negative mode) was used as an alternative method to analyze the variation of the Mn in the polyesters formed with the 10,16-DHPA. Chain length increase at 8 h to get a better distribution at 48 h (Mn 814 and Mw 1206).

### 2.2. Enzymatic Trans-Esterification Studies of the Methyl-10,16-Dihydroxyhexadecanoate

Only a few reports of transesterifications of long-chain aliphatic hydroxyacids have been reported in the literature [30,34]. The transesterification capability of lipases depends on the solvent used, and most of these reactions are carried out in toluene. However, other organic solvents such as hexane or acetonitrile can be used. The lipase-mediated reaction of methyl-10,16-DHHD was carried out mainly in two different solvents: acetonitrile and toluene. Both reactions gave similar molecular weight (Mw ~ 982 Da). However, when toluene was used, polyesters were obtained at a much shorter time (24 h, reaction time). The monomer consumption was similar to that investigated for the 10,16-DHPA polymerization. Initially, there was a fast consumption during the first 8 hours, which finally leveled off at about 95% conversion (Figure 1a). Even when the monomer shows a fast initial consumption, the molecular weights increase slowly to get a higher Mn at 24 h (Figure 1b). The higher solubility of the methyl ester in acetonitrile could have an opposite effect in the reaction because the substrate is more disperse than in toluene. Instead, the low solubility of the monomer in toluene could lead to the formation of micelles which are more available to the enzyme.

**Figure 2:**
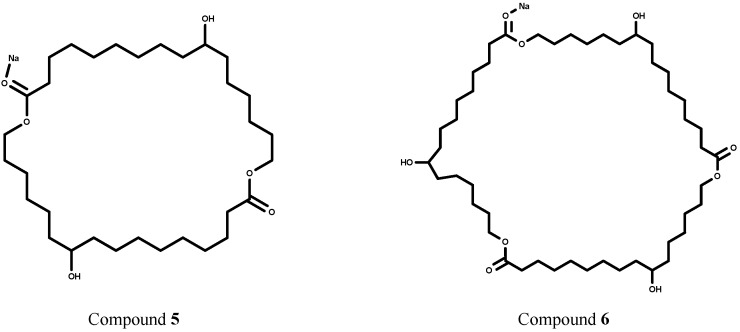
Macrocyclic compounds detected during the CALB-catalyzed polycondensations of methyl-10,16-DHHD in toluene at 60 °C (ESI-positive mode MS) compound **5** (MW 563) and compound **6** (MW 833).

Besides of linear oligomers, the ESI^+^ (positive ion) MS analysis showed the presence of a cyclic dimer (MW 563) and a cyclic trimer (MW 833) (Figure 2) formed during the methyl-10,16-DHHD lipase catalyzed reaction in toluene.

It is known that transesterification can lead to linear as well as macrocyclic products [37,38]. Macrocyclic compounds have been reported when transesterification occurs via back-biting reactions [29]. These transesterification reactions will broaden the molecular-weight distribution [37,38]. No macrocyclic compounds were detected in the lipase-catalyzed reactions of 10,16-DHPA.

The NMR analysis showed that the reaction was more efficient in toluene than in acetonitrile. When products were analyzed at the same concentration, the integration of the peak at δ 3.66 (^1^H-NMR) corresponding to the presence of the methyl ester group was smaller in the reaction with toluene than that with acetonitrile, indicating that conversion of the methyl-10,16-DHHD is better when toluene was used as a solvent.

Esterification of 10,16-DHPA was a little faster than transesterification of the methyl-10,16-DHHD under the same conditions (Figure 1a). The presence of a methyl group could have a small opposite effect in the chain growth in the lipase catalyzed reaction. However, the molecular weights were higher at 24 h in the transesterification lipase-catalyzed reaction (Figure 1b).

### 2.3. Characterization of the Products

#### 2.3.1. NMR analysis

**Figure 3:**
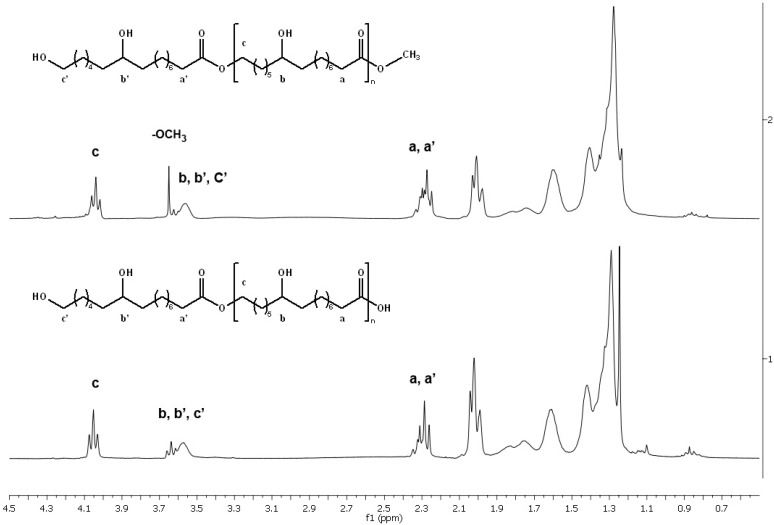
Comparison of the ^1^H-NMR spectra of methyl polyester obtained from methyl 10,16-dihydroxy-hexadecanoate (upper spectrum) and the polyester obtained from 10,16-dihydroxihexadecanoic acid (lower spectrum) in toluene at 24 h with CAL-B.

The lipase-catalyzed polyesterification reactions were followed first by HP-TLC (CHCl_3_-CH_3_OH, 95:5, v/v) until there was not change in the monomer on the plate (1, R_f_ 0.42; 2, R_f_ 0.54) and by HPLC to corroborate the reaction was completely done. The ^1^H-NMR spectrum of the polyester showed a triplet at δ 4.05 ppm, indicating the presence of an ester bond (-CH_2_-O-CO-, Figure 3). This was confirmed by the correlation in the HMBC of these protons with the carbonyl group at δ 173.9 ppm. There were no differences in the polymer NMR-spectra obtained from CAL-B, RM, TL, PCL or PPL.

Polyesters derived from the lipase-catalyzed transesterification of the methyl-10,16-DHHD showed similar NMR characteristics than those observed in the esterification products of 10,16-DHPA, a triplet at δ 4.05 ppm (-CH_2_-O-CO-) and a singlet at δ 3.66 ppm (-CO-O-CH_3_). NMR signals corresponding to macrocyclic compounds were difficult to analyze because these compounds were detected in low percentages (≈20% according to HPLC-ESI analysis) with respect to the presence of oligomers.

In both cases, esterification and transterification lipase-catalyzed reactions, the main products were linear polyesters. No cross-linked polyesters were detected.

#### 2.3.2. FT-IR Analysis

Typical IR spectra of monomers (10,16-DHPA and methyl-10,16-DHHD) [32] shows a large band from 2600 to 3300 cm^−1^ indicative of alcohol and carboxylic acid functional groups. Two intense sharp bands at 2918 and 2846 cm^−1^ result from C-H asymmetric and symmetric stretches within polymethylenic chains. An intense band at 1701 cm^−1^ for 10,16-DHPA and 1731 cm^−1^ for methyl-10,16-DHHD is attributed to C=O stretching. At 1410 cm^−1^ there is a band showing O-H bending in carboxylic acids. The band at 1259 cm^−1^ is due to C-O stretching in carboxylic acids while the band at 1067 cm^−1^ is C-O stretching in alcohols. Esters shows an important band at 1100 cm^−1^ attributed to C(=O)-O-C streching vibrations.

**Figure 4:**
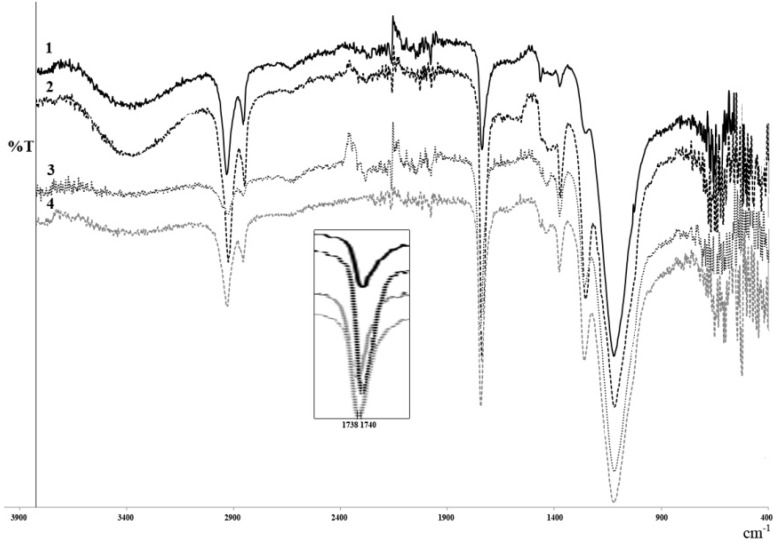
FTIR spectra of polyesters obtained with CAL-B and 10,16-DHPA in 2M2B(1) or toluene (2) and polymers obtained with CAL-B and methyl-10,16-DHHD in 2M2B (3) or toluene (4) at 24 h. The insert shows the small difference in the carbonyl group of both groups of polyesters.

The FTIR spectra of different polyesters obtained from CAL-B with 10,16-DHPA and methyl-10,16-DHHD in toluene and 2M2B are shown in Figure 4. In general, the spectra are very similar and, the majority of the bands correspond to an ester functional group. However, there are two small important differences between them. There is a band at 3365 cm^−1^ attributed to the presence of residual OH. This band is more intense in spectrum 1 and 2 from the polyester of the 10,16-DHPA obtained in 2M2B and toluene, respectively, than those obtained from methyl-10,16-DHHD due to the residual carboxylic group which is not present in spectra 3 and 4 (Figure 4). The analysis of some other bands corroborates the polyesterification of monomers: an intense band at 1738 cm^−1^ (spectra 3 and 4) and 1740 cm^−1^ (spectra 1 and 2) is attributed to C=O stretching in ester groups, and the asymmetric stretching vibration of the C-CO-O at 1172 cm^−1^ (Figure 4). These observations confirm the presence of a linear polyester group.

#### 2.3.3. MALDI-TOF MS and ESI MS Analysis

**Figure 5:**
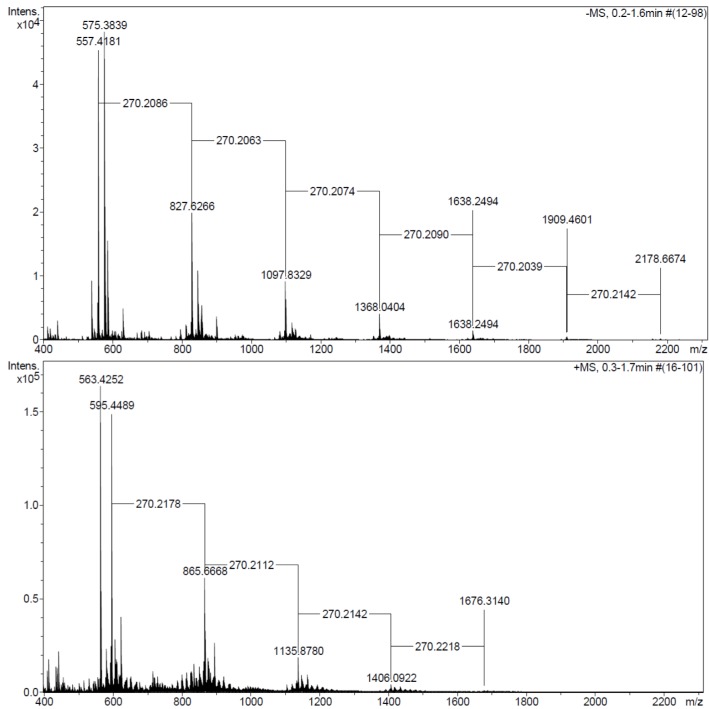
ESI MS Negative mode (upper spectrum) of the polyesters obtained from 10,16-DHPA and ESI-MS positive mode (lower spectrum) of the polyester obtained from methyl-10,16-DHHD, both reactions in toluene catalyzed by CAL-B at 60 °C in 24 h.

Polymeric distributions with the repeating unit of 270 Da were found in the region 500 to 3,500 *m/z* for the three soluble polyesters derived from the 10,16-DHPA (Figure 5). The repeating unit corresponds to 10,16-dihydroxyhexadecanoic acid losing a hydroxyl group:

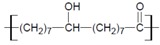



No signal was produced in the region from 3,000 to 50,000 *m/z*. There were no differences in the Mn observed among the products obtained from CAL-B and PCL, based on the detected distributions. The most abundant signal was at *m/z* 575 for the CAL-B and PCL samples. Weight average molecular weight (Mw) and number average molecular weight (Mn) could not be calculated for the other samples because only half of the distributions were recorded on the spectra. This may be caused by the signal suppression of matrix interferences. Those for sample CAL-B were Mw = 814 and Mn = 1,206 Da.

ESI+ MS (Positive mode) analysis was used to calculate the Mn and Mw of the polyesters derived from the methyl-10,16-DHHD lipase-catalyze reaction. Best results were observed when the CAL-B reaction was carried out in toluene, obtaining a Mw = 982 and Mn = 860 (Table 3).

#### 2.3.4. AFM Analysis

**Figure 6:**
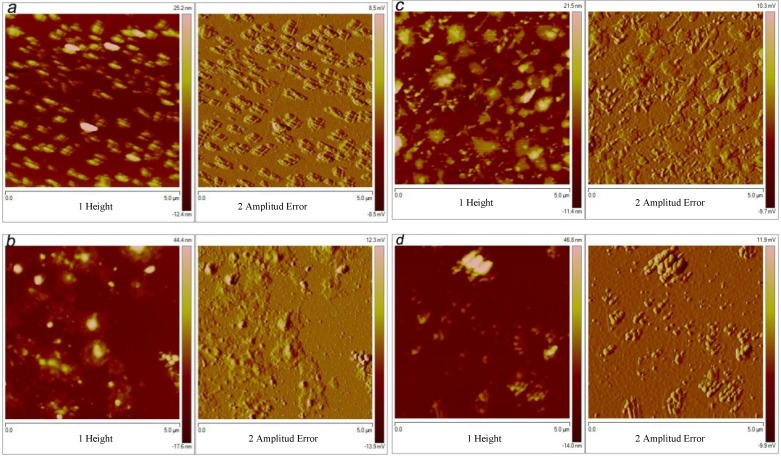
Atomic force microscopy tapping mode topographical images of polyesters obtained from 10,16-DHPA in a) toluene and b) 2M2B; and methyl-polyesters obtained from methyl-10,16-DHHD in c) toluene and d) 2M2B. All the reactions were done at 60 °C in 24 h. (Figures on the left side correspond to “height” images and figures on the right correspond to “amplitude error” images)

NMR, FT-IR and mass spectrometry analysis did not show clear differences between lipase-catalyzed polyesterification and transesterification reactions. However, the AFM data showed remarkable differences depending on the solvent used in the lipase-catalyzed reaction, even when all of them were prepared with the same solvent for the AFM analysis. The films formed on a watch glass after deposition of 5 μL of polyester solubilized in toluene showed differences in the structures of self-assembled layers (Figure 6). By means of AFM, the layer growth pattern on the surface can be extended to understand the formation of a robust molecule-substrate linkage and other interesting events controlled by weaker molecule-molecule interactions on these interesting nontoxic and biodegradable materials. The AFM images of the polyesters derived from the 10,16-DHPA in toluene, showed the formation of discrete islands of 350 µm^2^ as a maximum average area and approximately 51 nm as a maximum average height, covering about 30% of the film formed (Figure 6a). Curiously, the distribution of the islands showed a certain kind of order. Using the same reaction conditions, products from the transesterification of the methyl-10,16-DHHD in toluene showed a quite similar behavior (Figure 6c). In this case, more spread islands of 450–500 µm^2^ as a maximum average area and 34 nm as a maximum average height were observed. Unlike the structures observed in Figure 6a, these ones are interconnected covering a slightly lower area (26%).

Esterification and transesterification polyesters obtained from reactions in 2M2B showed remarkable differences with regard to those reactions prepared in toluene. For the lipase-catalyzed reaction of 10,16-DHPA, globular particles surrounded by halos were formed without apparent order (Figure 6b) of a maximum average area of 450–500 µm^2^ and approximately 68 nm as maximum average height, covering 24% of the total area. While the polyesterification of methyl-10,16-DHHD (Figure 6d) showed elongated islands (900 µm2) of a maximum average height detected of 61 nm, covering a very small area (10%–15%). The observed differences in the self-assembled layer could be related with the differences in the Mw of the polyesters (Table 3) and the presence of the macrocyclic compounds in the methyl-polyesters.

## 3. Experimental

### 3.1. Materials

Immobilized *Candida antarctica* lipase B (CAL-B, Novozym 435, specific activity 10,000 PLU/g), *Pseudomonas cepacia* lipase (PCL, Sigma-Aldrich, specific activity >900 U/g), Porcine pancreatic lipase (PPL, Sigma-Aldrich, specific activity >200 U/mg), immobilized *Rhizomucor miehei* lipase (Lipozyme RM IM, specific activity 150 IUN/g); immovilized *Thermomyces lanuginosus* lipase (Lipozyme TL IM, specific activity 250 IUN/g). Toluene, hexane, dimethylformamide (DMF), 2-methyl-2-butanol (2M2B) of reagent grade were purchased from Merck (Naucalpan de Juárez, Edo. de México) and used without further purification.

### 3.2. Methods

Polyesters obtained from the lipase-catalyzed reactions were characterized by ^1^H- and ^13^C-NMR (Varian NMR System, 500 MHz). The NMR spectra were recorded in deuterated chloroform (CDCl_3_) or methanol (CH_3_OD). FTIR Spectra were recorded with a BOMEM 157 FTIR spectrometer equipped with a deuterated triglycine sulfate (DTGS) detector. The instrument was under continuous dry air purge to eliminate atmospheric water vapor. The spectra were recorded in the region of 4000–400 cm^−1^. Molecular mass and molecular mass distributions were determined by MALDI-TOF techniques. Mass spectra were obtained on a Bruker Autoflex Matrix-Assisted Laser Desorption/Ionization Time-of-Flight Mass Spectrometer. Soluble samples were dissolved in chloroform to concentrations of approximately 10 mg/mL. The samples were then mixed with different matrices in the volume ratio 4:1 to 5:1. MALDI conditions were varied widely to obtain optimal spectra by adjusting laser power (30−90%), reflectron voltage gain (12×–60×), and pulsed ion extraction (10–300 ns). The best results were obtained using CHCA organic salt as a matrix. Polymeric distributions with the repeating unit of 270 Da were found in the region 500 to 3,500 *m/z* for the soluble polymers. Electrospray Ionization (ESI) analysis was done on a Bruker micrOTOF-Q II (Bruker Daltonics, Bremen, Germany). Samples were dissolved in methanol and were injected directly to the spectrometer. The polymer related peaks were found in positive and negative ion mode [ESI]. The capillary potential was −4.5 kV, the dry gas temperature 200 °C and the drying gas flow 4 L/min. Total ion chromatograms from *m/z* 500 to 3,000 were obtained. MS data were processed using PolyTools 1.0 (Bruker Daltonics). Atomic Force Microscopy (AFM) was used to analyze polyesters films prepared using a solution of 1 mg of the polyester dissolved in 1 mL of toluene. The atomic force microscope used was a Veeco diMultimode V operating in air room temperature equipped with a medium-range scanner (15 × 15 µm^2^ X-Y and 2.3 µm^2^). Reproducibility of AFM images were ensured by scanning in at least three points of the surface of each film. AFM was operated in tapping mode at a scan rate of 1Hz using RTESP Model tips. Image analysis was done using the NanoScope Analysis 1.2 Software by Veeco to obtain the maximum average area and the maximum average height by analyzing three images for each case.

### 3.3. Isolation of 10,16-Dihydroxyhexadecanoic Acid (10,16-DHPA, **1**)

10,16-DHPA (**1**) was obtained by depolymerization of tomato cutin as described elsewhere [20,25]. Identification of the monomer was done by NMR spectrometry and MS analysis and the data was compared with those previously reported [32,33]. Yellow pale powder. ^1^H-NMR (500 MHz, CH_3_OD) δ ppm 3.63 (m, 4H) C(OH)H-10 and C(OH)H_2_-16, 2.27 (t, *J* = 7.52 Hz, 2H) CH_2_-2, 1.60–1.22 (m) CH_2_-3-9 and CH_2_-11-15. ^13^C-NMR (CH_3_OD) δ 177.68 (CO); 72.40 (C-10); 63.00 (C-16). EI-MS (*m/z*): 289 [M+H]^+^, 271 [M-H_2_O]^+^, 253 [M-2H_2_O]^+^, 130 [C_8_H_18_O_2_-OH]^+^ [33].

### 3.4. Preparation of Methyl 10,16-Dihydroxyhexadecanoate (Methyl-10,16-DHHD, **2**)

HCl (0.05 mL) was added to a stirred solution of compound **1** (0.86 mM in absolute methanol). The mixture was allowed to react overnight at room temperature, and then evaporated at reduced pressure. The remaining HCl was co-evaporated with methanol, and the product **2** was recovered without further purification from CH_2_Cl_2_. Yellow pale powder. ^1^H-NMR (500 MHz, CHCl_3_) δ ppm 3.66 (s, 3H) CH_3_-O, 3.64 (m, 4H) C(OH)H-10 and C(OH)H_2_-16, 2.30 (t, *J* = 7.52 Hz, 2H) CH_2_-2, 1.60-1.22 (m) CH_2_-3-9 and CH_2_-11-15. ^13^C-NMR (CHCl_3_) δ 179.12 (CO); 72.20 (C-10); 63.12 (C-16); 51.64 (-OCH_3_). FAB-MS (*m/z*): 302 [M]^+^, 288 [M+H-15]^+^ [33,35].

### 3.5. Polyesterification of 10,16-DHPA and Trans-Esterification of Methyl-10,16-DHHD

10,16-DHPA (**1**, 40 mg, 130 mM) or methyl-10,16-DHHD (**2**, 40 mg, 132 mM) (Scheme 1) and lipase (10 wt % of the total monomer wt) were added to capped reaction vials (5 mL). Toluene, 2-methyl-2-butanol (2M2B), dimethyl formamide (DMF), acetonitrile or hexane (400 μL) was used as solvent in different reactions, and the reactions were placed in a shaking incubator (VorTemp™ 1550). The vials were shaken at 120 rpm for 48 h at 60 or 75 °C. Reactions without addition of lipase were used as controls. In a different series of experiments, activated molecular sieves (20 mg Merck 4 Å) were added to the vials to remove water. Once the reaction was complete, the reaction mixture was filtered to remove the enzyme and the solvent was concentrated under reduced vacuum. Polyesters were extracted with chloroform and were characterized by NMR, MS, FT-IR and AFM analysis.

*Polyesters from 10,16-DHPA* (**3**), ^1^H-NMR (500 MHz, CDCl_3_) δ ppm 4.05 (t, 2H) -O-CH_2_-, 3.63 (m, 4H) C(OH)H-b, C(OH)H-b′, C(OH)H-c and C(OH)H-c′, 2.27 (t, J = 7.52 Hz, 2H) CH_2_-a and CH_2_-a′, 1.60–1.22 (m) –CH_2_-. ^13^C-NMR (125 MHz, CDCl_3_) δ 177.68 (CO); 72.40 (C-b and C-b′); 63.00 (C-c and C-c′).

*Methyl-polyesters from methyl-10,16-DHHD* (**4**), ^1^H-NMR (500 MHz, CDCl_3_) δ ppm 4.04 (t, 2H) -O-CH_2_-, 3.65 (s, 3H) CH3-O, 3.57 (m, 4H) C(OH)H-b, C(OH)H-b′, C(OH)H-c and C(OH)H-c′, 2.28 (t, *J* = 7.52 Hz, 2H) CH_2_-a and CH_2_-a′, 1.60–1.22 (m) -CH_2_-. ^13^C-NMR (125 MHz, CDCl_3_) δ 174.56 (CO); 72.10 (C-b and C-b′); 64.60 (C-c and C-c′), 51.60 (-OCH_3_).

### 3.6. Conversion of 10,16-DHPA and Methyl-10,16-DHHD by CAL-B

In order to know the conversion of 10,16-DHPA and methyl-10,16-DHHD by CAL-B *versus* time, 40 mg of 10,16-DHPA (1) or methyl-10,16-DHHD (2) and 10%-by-wt CAL-B relative to monomer were added to capped reaction vials (5 mL). Toluene (1 mL) was used as solvent and the sample was placed in a shaking incubator (VorTemp™ 1550). The vials were shaken at 120 rpm at 60 °C and samples of the reaction were taken at 0, 1, 4, 8, 12, 24 and 48 h to be analyzed by an HPLC system. The reactions were terminated by adding excess of cold chloroform, stirring for 15 min, and removing the enzyme by filtration (glass-fritted filter, medium porosity). These non-fractionated products (no precipitated) were analyzed by HPLC-ELSD and characterized by ^1^H-NMR, ESI MS and MALDI-TOF-MS to determine their molecular-weight distribution and to analyze the different species generated.

### 3.7. HPLC Analysis

10,16-Dihydroxyhexadecanoic acid was analyzed using an VARIAN HPLC system with an evaporative light scattering (ELSD) detector (Alltech, Deerfield, IL, USA). Analyses of the samples were done using a 0.5 mL/min flow rate, in a isocratic system of a mixture of acetonitrile-water (75:25, v/v) and a C_18_ Symmetry column (4.6 mm × 75 mm, ID 3.5 µm). The volume of the injected sample was 20 mL. ELSD detection used nitrogen gas pressurized at 26.6 psi and flowing at 1.8 SLM and a drift tube temperature of 30 °C

## 4. Conclusions

Functionalizable polyesters bearing free hydroxyl groups were designed and successfully synthesized in a simple lipase-catalyzed reaction from 10,16-DHPA (isolated from tomato cuticle) and methyl-DHHD. All the polyesters were linear oligomers according with the NMR, FT-IR, ESI MS and MAL-TOF MS analysis and there was no evidence of any branched polyester. Even when the oligomers showed a relatively small molecular weight, the presence of free hydroxyl groups make them interesting compounds to design higher functionalizable polyesters. We are currently investigating bulk polymerizations and different interactions between monomers such as glycerol and 10,16-DHPA to expand the application of these natural-derived polymers. We expect this new design platform will lead to a diverse family of biodegradable and bioactive polymers with versatile mechanical, physical, chemical, and biological properties tailored for a wide range of biomedical or industrial applications.
